# Comparative Analysis of Age-Associated Changes in Meibum Composition, Distribution, and Function in Mice With Altered Hyaluronan Expression

**DOI:** 10.1167/iovs.66.9.72

**Published:** 2025-07-30

**Authors:** Sudhir Verma, Paola A. Guevara Montoya, Mingxia Sun, Sudan Puri, Seher Yuksel, Amber Wilkerson, Tarsis Ferreira Gesteira, Igor A. Butovich, Vivien J. Coulson-Thomas

**Affiliations:** 1 College of Optometry, University of Houston, Houston, Texas, United States; 2 Department of Zoology, Deen Dayal Upadhyaya College, University of Delhi, Delhi, India; 3 Ophthalmology, The University of Texas Southwestern Medical Center, Dallas, Texas, United States

**Keywords:** meibomian gland, hyaluronan, aging, dry eye, ocular surface, corneal opacity

## Abstract

**Purpose:**

Mice lacking hyaluronan (HA) synthase 1 and 3 (*Has1^−/−^; Has3^−/−^* mice) are resistant to meibomian gland (MG) atrophy and dropout. Herein, we characterized the composition and distribution of meibum in *Has1^−/−^;* *Has3^−/−^* mice as they age and verified whether they are protected from developing dry eye disease (DED).

**Methods:**

Tarsal plates from wild-type (wt) and *Has1^−/−^; Has3^−/−^* mice were isolated, and meibum lipid composition and the distribution of meibum were analyzed by liquid chromatography–mass spectrometry and whole-mount Sudan IV staining, respectively. The expression of biosynthetic enzymes for major lipid populations was analyzed by real-time PCR. The efficacy of the meibum at stabilizing the tear film was assessed using the benzalkonium chloride dry eye model, and DED-related symptoms were assessed in aged mice.

**Results:**

*Has1^−/−^* *;* *Has3^−/−^* mice do not present a decrease in meibum production with aging and express significantly more meibum at all ages when compared to wt*. Has1^−/−^;* *Has3^−/−^* mice show significantly fewer age-related changes to meibum lipid composition when compared to wt mice. Overall, *Has1^−/−^;* *Has3^−/−^* mice have significantly less corneal opacity and epithelial erosions in aged mice when compared to wt mice. *Has1^−/−^;* *Has3^−/−^* mice are protected from developing DED when compared to wt mice.

**Conclusions:**

Our findings show that in addition to preventing MG atrophy, the overexpression of an HA-rich matrix surrounding the MG supports the production of healthy meibum that can successfully stabilize the tear film and protect the ocular surface from the effects of age-related meibomian gland dysfunction and dry eye symptoms.

Meibomian glands (MGs) are serially arranged, long, linear structures present in the tarsal plates within eyelids that lie perpendicular to the eyelid margin.^1^ Each gland is made up of multiple secretory acini lined by meibum-producing secretory cells, the meibocytes. The acini are connected to a central duct via connecting ductules, which collect the meibum from the acini and deliver it to an excretory duct that opens to the lid margin.^2^ The holocrine secretion from MGs, named meibum, stabilizes the tear film, prevents tear evaporation, and protects the ocular surface.^3^ Dry eye disease (DED) is a significant public health concern affecting millions of people worldwide, with an estimated global prevalence ranging from 5% to 50%, depending on different geographical regions.^1^^,^^4^^,^^5^ Patients with DED typically have symptoms that include ocular surface irritation, burning sensation, dryness, foreign body sensation, photophobia, blurred vision, and pain, and in more severe cases, DED can progress to corneal inflammation, chronic epithelial erosions, infection, extreme pain, and corneal scarring, which ultimately lead to impaired vision.^6^ Studies have shown that approximately 85% of cases of DED are caused by meibomian gland dysfunction (MGD).^7^ Unfortunately, currently, no effective treatments exist for preventing or reversing MGD, and patients rely on palliative care to relieve their symptoms.^8^ The etiology of MGD remains unknown; thus, further research is needed in increase our understanding on the pathophysiology of MGD so that better therapeutic approaches can be developed.

MGD is a widespread eyelid disorder that encompasses structural and functional abnormalities of the MGs and is recognized in three forms: obstructive MGD, hyposecretory MGD, and hypersecretory MGD, with the first form considered the most common.^1^^,^^9^^,^^10^ Various studies have shown that in obstructive MGD, hyperkeratinization of MG ducts leads to ductal occlusion and plugging of orifices, causing cystic dilation of the ducts and atrophy of acini, which progresses to MG dropout.^9^^,^^11^^–^^15^ Various risk factors have been associated with MGD, including hormonal imbalances, diet, prolonged contact lens wear, excessive use of makeup, eyelid tattooing, *Demodex folliculorum* infestation, and certain systemic conditions, such as Sjögren syndrome, rosacea, lupus, psoriasis, rheumatoid arthritis, Stevens-Johnson syndrome (SJS), and hypertension.^16^ However, to date, aging is considered the major risk factor for MGD, leading to age-related MGD (ARMGD). With age, MGs undergo various structural and functional changes, including atrophy and dropout; however, the pathobiology underlying MG atrophy and dropout remains unknown.^9^^,^^16^^,^^17^ Parfitt and colleagues^18^ previously showed that aged mice present a significant decrease in total gland volume, which was correlated with a decrease in basal cell proliferation and a loss of progenitor cells, which were not associated with ductal hyperkeratinization. These structural changes observed in aged murine MGs were consistent with changes observed in aged human MGs.^19^ Studies have also shown that the amount,^20^ composition, and viscosity^21^ of meibum change with aging, as seen in both humans and mice.^15^ Thus, significant data have shown that MGs undergo significant anatomic and functional alterations with age.

Our group has previously shown that a highly organized hyaluronan (HA)–rich extracellular matrix exists surrounding the MG and in the tarsal plate.^22^^,^^23^ Furthermore, we found that this HA-rich matrix regulates MG morphogenesis and homeostasis.^22^ HA, a major component of the extracellular matrix, is a nonsulfated glycosaminoglycan composed of a repeating disaccharide unit composed of glucuronic acid and N-acetylglucosamine.^24^^–^^26^ In tissues, HA is biosynthesized by enzymes, HA synthases (HASs), of which mammals have three isoforms: HAS1, HAS2, and HAS3.^22^^,^^27^^,^^28^ HAS2 is ubiquitous, whereas HAS1 and HAS3 have a specific spatial and temporal distribution throughout development, homeostasis, and aging.^29^^,^^30^ Previously, our group showed that all three isoforms are expressed in the tarsal plate and surrounding MGs, with *Has2* being the major isoform expressed. Furthermore, we showed that *Has1* and *Has3 null* (*Has1^−/−^; Has3^−/−^*) mice upregulate *Has2* expression, and consequently, these mice express increased levels of HA surrounding MGs and in the tarsal plate.^22^^,^^23^ Recently, we reported that with age, this HA-rich matrix surrounding MGs is lost in wild-type (wt) mice, contributing toward ARMGD.^23^ In contrast, *Has1^−/−^; Has3^−/−^* mice, which express higher levels of HA and do not present a decrease in HA with aging, are resistant to developing MG atrophy and thus do not present ARMGD.^23^ However, whether these enlarged glands produce healthy meibum that can effectively stabilize the tear film and protect against DED remains unknown. In the present study, we characterized the composition and distribution of meibum within the MGs of *Has1^−/−^; Has3^−/−^* mice and wt mice as they age. We further investigated whether the enlarged MGs in *Has1^−/−^; Has3^−/−^* mice produce healthy meibum that can stabilize the tear film and protect the ocular surface from DED damage.

## Materials and Methods

### Animals

C57BL/6J mice (Strain 000664 ; The Jackson Laboratory, Bar Harbor, ME, USA), hereafter referred to as wt mice, and *Has1* and *Has3* double *null* mice, hereafter referred to as *Has1^−/−^; Has3^−/−^* mice, were used. Transgene alleles were identified by PCR genotyping with tail DNA for all mice in the *Has1^−/−^;* *Has3^−/−^* mouse colony. Tissues were collected at 8 weeks (adult), 1 year (aging), and 2 years (aged) of age. Both male and female mice were included in the study in all experimental groups. In total, 186 mice were used equally distributed between wt and *Has1^−/−^; Has3^−/−^* mice. Specifically, 52 adult, 52 aging, and 82 aged animals were used for different experiments in this study. All mice were housed in a temperature-controlled room with an automatic 12-hour light/dark cycle at the animal facility of the University of Houston. Aging mice were provided with additional enrichment, including Shepherd Shack papers (Backless Shack, SS-2; Lab Supply, Fort Worth, TX, USA) from 6 months. All experimental procedures for handling the mice were previously approved by the Institutional Animal Care and Use Committee at the University of Houston under protocol 16-044. Animal care and use conformed to the ARVO Statement for the Use of Animals in Ophthalmic and Vision Research. All methods are reported in accordance with ARRIVE guidelines.^31^

### Stereomicroscope Imaging of the Ocular Surface and Meibomian Glands

For imaging the ocular surface, mice were anesthetized with 80 mg ketamine (Dechra Veterinary Products, Overland Park, KS, USA) and 10 mg xylazine (Dechra Veterinary Products) per kilogram of body weight, injected intraperitoneally. After confirming the depth of anesthesia, the mouse was placed under a stereo zoom microscope (Discovery.V12 Modular Stereo Microscope; Carl Zeiss Microscopy LLC, Oberkochen, Germany) and imaged under white light for detecting corneal opacity and ring light for detecting the corneal smoothness/irregularity (VisiLED Ring Light; Carl Zeiss Microscopy LLC). The corneal surface was stained with 2 µL fluorescein stain (1 mg/ mL) (GloStrips; Amcon Labs, St. Louis, MO, USA), washed with PBS, and imaged under a GFP filter (X-cite 120Q; Excelitas Technologies, Waltham, MA, USA) to identify corneal epithelial defects. The same imaging settings were used for imaging all mice.

### Calculation of Corneal Fluorescein Staining Score and Corneal Opacity Score

Images obtained from the ocular surface were analyzed and objectively ranked for corneal fluorescein staining score (CFSS) and corneal opacity score (COS) by inspecting digital images acquired by two experienced investigators (S.V. and V.J.C.-T.) in a masked manner. For the COS, the following grading system was used: 0, clear cornea; 1, slight opacity throughout the cornea, with details of the iris still visible; 2, severe opacity covering at least one quadrant of the cornea; 3, severe opacity covering at least two quadrants of the cornea; 4, severe opacity covering at least three quadrants of the cornea; and 5, severe opacity covering the entire cornea. For the CFSS, the following grading system was used: 0, no staining; 1, minimal staining with only a few punctate spots; 2, mild staining with moderate punctate spots covering about 25% of the cornea; 3, moderate staining with moderate punctate spots covering about 50% of the cornea; 4, severe staining with moderate punctate spots covering about 75% of the cornea; and very severe, dense staining covering the whole cornea.

### Calculation of Ring Light Circularity

An in-house MATLAB script was developed and used to calculate the circularity of the image of the ring light reflected from the ocular surface. Briefly, the ring light images were binarized using Otsu's threshold. The mean of bright pixel locations was defined as the center, and thereafter, sampling at 1-degree intervals was made radially from the center. The mean position of the innermost bright pixels refined the center location. Sampling radially from the refined center, the outermost bright pixels defined the outer perimeter of the reflected region. The circularity (c) of the point-to-point perimeter of these pixels was computed as c = 4π * a/p^2^, where a is the enclosed area and p is the perimeter. The circularity of the reflected ring light images obtained from the cornea of aged wt and *Has1^−/−^; Has3^−/−^* mice was plotted as a box plot.

### Collection and Processing of Eyelids

Mice were euthanized by CO_2_ inhalation followed by cervical dislocation, and eyelids were collected. The eyelids to be used for isolating MGs for meibum collection were stored immediately at −80°C in a deep freezer for further processing. The eyelids designated for Sudan IV staining were immediately mounted as flat mounts after removing excessive skin and hair and imaged under a stereomicroscope using white light (Discovery.V12; Carl Zeiss Microscopy LLC). Thereafter, eyelids were unmounted and fixed in 4% buffered paraformaldehyde at 4°C for 24 hours and then transferred to PBS. For quantitative real-time PCR analysis, after flat-mount imaging, tarsal plates were immediately isolated and stored at −80°C. Approximate time of collection to fixation was ∼4 minutes, and the approximate time of collection to storage at −80°C for messenger RNA (mRNA) isolation was ∼7 minutes.

### Stereomicroscope Imaging of Whole–Mounted Meibomian Gland

The isolated eyelids were trimmed to reveal the MGs under a dissecting microscope (Leica S9E; Leica Microsystems, Deerfield, IL, USA). Excess connective tissue, skin, and hair were removed using a pair of microsurgical scissors to enable exposure of the lateral and medial canthus. The eyelids were then flat- mounted between two microscope slides and imaged with the conjunctival surface facing upward. Flat-mount images of the MGs were acquired using a stereomicroscope Discovery.V12 with an Axiocam 503 color camera (Carl Zeiss Microscopy LLC ) under white light.

### Quantification of the MG Area

The total MG area was quantified using ilastik (https ://www.ilastik.org/), as previously shown,^23^ for male and female 8-week, 1-year, and 2-year wt and *Has1^−/−^; Has3^−/−^* mice. Each point of the graph indicates an individual mouse.

### Sudan IV Staining

For staining the meibum lipids in eyelid whole mounts, an established protocol by Hamada et al.^32^ was used with some modifications. Briefly, the eyelids were fixed in 4% buffered paraformaldehyde for 24 hours at 4°C, washed with PBS for 15 minutes each, and bleached with 3% (v/v) hydrogen peroxide at 37°C for 24 hours to remove pigments. The samples were fixed again with 4% buffered paraformaldehyde for 24 hours at 4°C and immersed in 50% ethanol for 10 minutes and 70% ethanol for 20 minutes at room temperature (RT). Thereafter, the samples were stained with Sudan IV–saturated 70% ethanol solution containing 2% NaOH for 24 hours with gentle rotation at room temperature, followed by extensive washing with 70% ethanol at RT. Samples were then cleared by incubating with 25% N, N, N′, N′-Tetrakis-(2-hydroxypropyl) ethylenediamine in glycerol overnight, followed by mounting in glycerin. Flat-mount images were taken under a light-field stereomicroscope and analyzed using ImageJ (National Institutes of Health, Bethesda, MD, USA).

### Meibum Extraction and Analysis Using Liquid Chromatography–Mass Spectrometry

Meibum was extracted following a previously described protocol based on prior work that showed that physical expression of meibum from the tarsal plate does not provide quantitative data that are representative of meibum expressed by MGs.^33^ For meibum extraction, a total of 24 mice were used (12 wt and 12 *Has1^−/−^; Has3^−/−^*): 4 mice (2 males and 2 females) each of wt and *Has1^−/−^; Has3^−/−^* genotype of adult, aging, and aged groups. Briefly, tarsal plates were surgically excised from the upper and lower eyelids of individual euthanized animals by an experienced surgeon and placed in glass vials with 1 mL chloroform/methanol at a 2:1 (v/v) solvent mixture. The lipids were extracted thrice each time with ∼1 mL solvent, the three extracts were pooled and brought to dryness under a stream of ultra-high-purity nitrogen at 36°C, and the residue was re-dissolved in 1 mL isopropanol. The samples were stored in sealed vials at −80°C until analysis.

Lipidomic analyses were performed using ultra-high -pressure liquid chromatography–high- resolution mass spectrometry (LC-MS).^33^ All LC-MS equipment and software were from Waters Corp. (Milford, MA, USA). The analytes were separated on a C8 BEH Acquity column (1.7 µm, 2 × 100 mm) for total lipid analysis or a C18 BEH Acquity column (1.7 µm, 1 × 100 mm) for individual lipid analyses. An Acquity M-Class LC system was used. The lipids were detected using a Synapt G2-Si (Milford, MA, USA) time-of-flight mass spectrometer operated in high-resolution atmospheric pressure chemical ionization (APCI) positive ion mode (PIM). The mass-to-charge (*m/*z) ratios of analytes were detected with better than 5-mDa accuracy. The structures of the major lipid analytes were confirmed in MS/MS experiments, as described earlier.^34^ Wax esters (WEs) were detected as (M + H)^+^ adducts, while free cholesterol formed (M + H − H_2_O)^+^ ions. All cholesteryl esters (CEs) were observed as (M + H − fatty acid)^+^ adducts.

**Figure 1.**
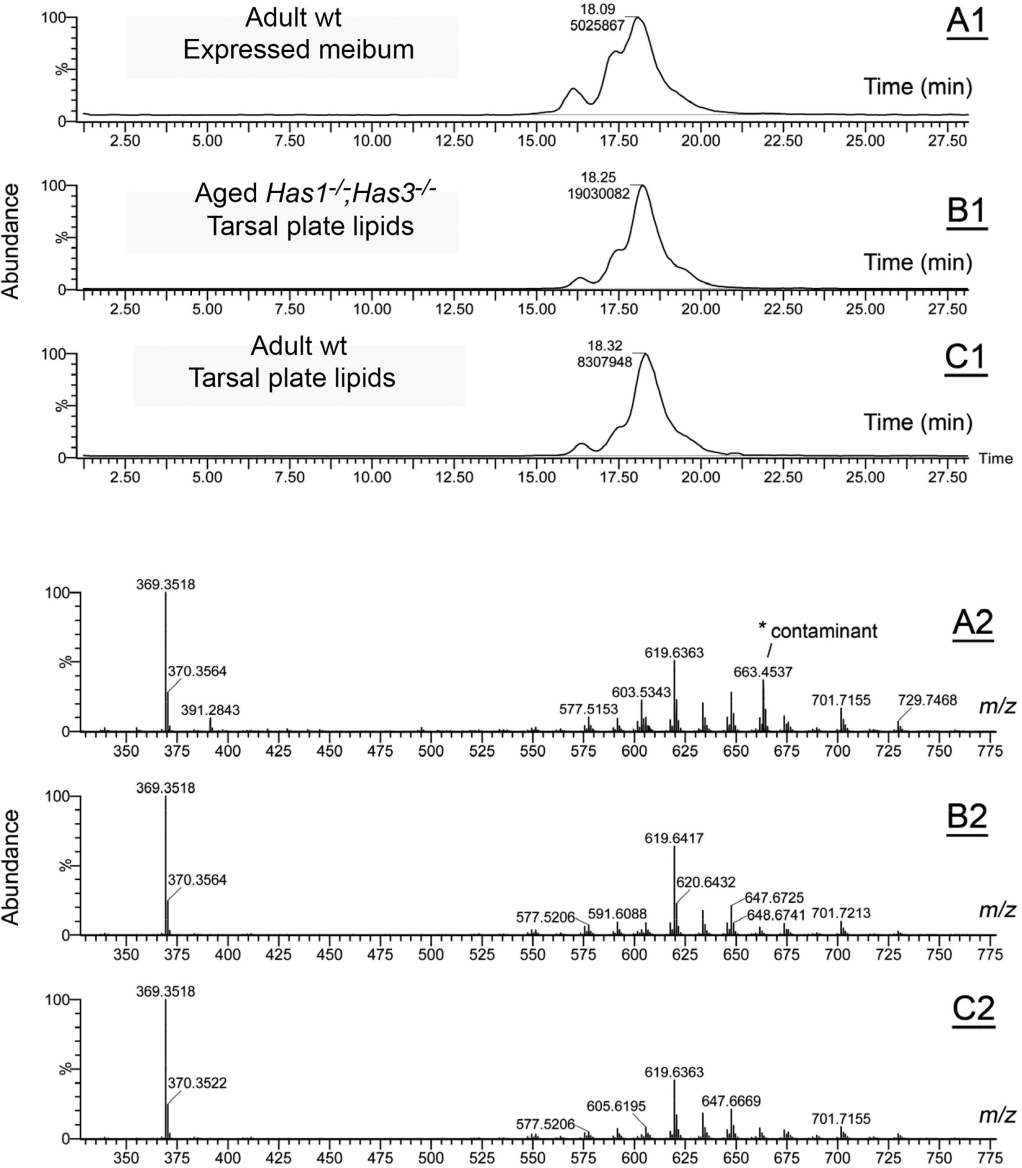
Representative isocratic reverse -phase C8 chromatograms and observation mass spectra of mouse MG lipids recorded under atmospheric pressure chemical ionization positive ion mode. Total ion chromatogram (**A1**) and observation mass spectrum (**A2**) of a sample of meibum that was physically expressed from the eyelids of an adult wt male mouse. The LC-MS peak is labeled as Retention Time (upper figure; in minutes) and Peak Area (lower number; in arbitrary units). A known chemical contamination with an *m/z* value of 663.4537 is labeled with an *asterisk*. Total ion chromatogram (**B1**) and observation mass spectrum (**B2**) of a sample of meibum extracted with organic solvents from the eyelids of an aged *Has1^–/–^Has3* *^–^^/^^–^* female mouse. Total ion chromatogram (**C1**) and observation mass spectrum (**C2**) of a sample of meibum extracted from the eyelids of a wild-type, 2- month-old male mouse with organic solvents.

Both targeted and untargeted lipidomic analyses of *wt* and *Has1^−/−^; Has3^−/−^* mice were performed. Total lipid production was estimated by integrating total ion chromatograms obtained on the C8 column in isocratic mode under identical conditions (such as number of tarsal plates per sample, total sample volume, injected volume, LC-MS running conditions).^35^ Representative chromatograms and mass spectra of wt and *Has1^−/−^; Has3^−/−^* mouse meibum are shown in Figure 1. Note extremely high similarities between expressed meibum (Figs. 1A1, 1B1), as well as extracted tarsal plate lipids of aged female knockout mice (Figs. 1B1, 1B2) and young male wild-type mice (Figs. 1C1, 1C2), but higher lipid production in the aged sample (peaks areas of 8.3 × 106 vs. 1.9 × 106 arbitrary units, correspondingly). As digital expression of meibum from the eyelids of mice cannot be used for its quantitation, the tarsal plate lipid extracts were used instead. For untargeted analyses, the raw C8 LC-MS APCI PIM files were initially processed with the Progenesis QI software package (version 2.3; from Nonlinear Dynamics/Waters Corp., Milford, MA, USA) and EZinfo software package (version 3.0.3; from Umetrics AB/Waters Corp., Milford, MA, USA). First, the data for individual lipids were normalized against the total ion count in each tested sample, which is a standard procedure in Progenesis QI software, and then exported to EZinfo software. Once transferred to EZinfo, the data were initially overviewed using its principal component analysis (PCA) template and then reanalyzed for intergroup differences using its partial least squares discriminant analysis (PLS-DA) routine with the following model details: six conditions (2 genotypes *wt* and *Has1^−/−^; Has3^−/−^* and 3 age groups: adult [8 weeks old], aging [1 year old], and aged [2 years old ]), Pareto scaling, no transformation, and 330 variables found, none excluded. Note that both PCA and PLS-DA approaches use normalization procedures and provide information on the relative balance of lipids within the study samples and groups, while the total amount of lipids in each sample can be estimated from integrated total ion chromatograms of individual samples, as described earlier.^35^ Thus, to derive the total amounts of individual lipids in the samples of different types, one needs to account for the total lipid production as well.

For targeted lipid analysis, extracted ion chromatograms of major lipid species were generated from the raw LC-MS data files using the mass spectra of meibum samples (Fig. 1A) and the theoretical *m/z* values of major analytes (Supplementary Table S1). The corresponding LC-MS peak areas were used to compare wt and *Has1^−/−^; Has3^−/−^* specimens (Fig. 1B).

### RNA Extraction and Real–Time PCR Analysis

Tarsal plates isolated from wt and *Has1^−/−^; Has3^−/−^* mice at 8 weeks, 1 year, and 2 years of age were processed for quantitative RT-PCR to investigate the expression of enzymes involved in meibum biosynthesis. The enzymes selected were fatty acyl-CoA reductases 1 and 2, acyl-CoA wax alcohol acyltransferases 1 and 2, and acyl-coenzyme A:cholesterol acyltransferase/ sterol O-acyltransferase 1 and 2, along with two housekeeping genes, β-actin and Gapdh. Primer sequences used are listed in Table. Total mRNA was isolated from the tarsal plates using Trizol Reagent (Invitrogen, Carlsbad, CA, USA), according to the manufacturer's instructions. RNA concentration and purity were determined using a nanodrop spectrophotometer (NanoDrop 2000; ThermoFisher Scientific, Waltham, MA, USA) at 260 and 280 nm. The 2 µg total RNA was used for synthesizing first-strand cDNA with the high-capacity cDNA reverse transcription kit (Applied Biosystems, Waltham, MA, USA), according to the manufacturer's instructions. The expression levels of genes of interest were analyzed by quantitative RT-PCR using the Powerup SYBR Green Master Mix kit (Applied Biosystems) using a BIORAD (Hercules, CA, USA) CFX Connect Real-time System. Amplification consisted of an activation cycle of 95°C for 10 minutes, followed by 40 cycles of 95°C for 15 seconds and 60°C for 1 minute. Relative quantification of the expression levels was carried out against the housekeeping genes Gapdh and β-actin (Actb), using both the *2^−^^ΔCt^* and *2^–^^ΔΔCt^* method. The specificity of the amplified products was analyzed through dissociation curves generated by the equipment, yielding single peaks and gel electrophoresis of amplified products. At least six samples were pooled per experimental point.

**Table.** Sequence of the Primers Used for Quantitative RT-PCR

Gene	Enzyme	Forward (5′-3′)	Reverse (5′-3′)
*Far1*	Fatty acyl-CoA reductase 1	GATAATGTCAATATGTTAATGAACC	TCAGTATCTCATAGTGCTGGATGCTCG
*Far2*	Fatty acyl-CoA reductase 2	TCCATGCTGGAGTATTTCATCAACC	TTGAACAAGGGACAAATGAAGAACC
*Awat1*	Acyl-CoA wax alcohol acyltransferase 1	GGAGAAACAGAGGTATATGACCAGG	TCACAAGAATATCAGCTTCTGGGTGTTGG
*Awat2*	Acyl-CoA wax alcohol acyltransferase 2	GGAGAGACAGACCTCTATGACCAGC	TCAAACTATCACCAGCTCCTGGGTC
*Acat1/ Soat1*	Acyl-coenzyme A:cholesterol acyltransferase 1/ sterol O-acyltransferase 1	GCCGTCTTCGCCCTGTCGGCTGTGG	CTAAAACACGTACCGACAAGTCCAGG
*Acat2/ Soat2*	Acyl-coenzyme A:cholesterol acyltransferase 2/ sterol O-acyltransferase 2	GAGATTGTGCCAGTGCTGGTGT	GTGACAGTTCCTGTCCCATCAG
*Gapdh*	Glyceraldehyde-3-phosphate dehydrogenase	AGGTTGTCTCCTGCGACTTCA	CAGGAAATGAGCTTGACAAAGTTG
*Actb*	—	GAGACCTTCAACACCCCAGC	CCTCGTAGATGGGCACAGTGTG

### Benzalkonium Chloride Dry Eye Model

Wt and *Has1^−/−^; Has3^−/−^* mice at 8 weeks were gently but securely restrained, and 10 µL of 0.1% benzalkonium chloride (BAC) was placed onto the ocular surface of both eyes twice daily for 21 days. Mice were monitored daily for signs of discomfort. At the start of the experiment and at the end of each week, the ocular surface was clinically assessed and imaged under a light-field stereomicroscope. Five males and five females were used for each experimental group, and since no sex differences were noted, data were aggregated by genotype.

### Tear Breakup Time

Tear film stability was measured using tear breakup time. For such, 1 µL of 0.1% sodium fluorescein was administered into the ocular surface, followed by three forced blinks. After the third blink, the time to the first observation of a break in the tear film under a slit-lamp microscope was recorded in seconds. The tear breakup time (TBUT) procedure was repeated three times per animal for each time point and the average TBUT presented. Epithelial damage was measured using corneal fluorescein staining. Ninety seconds after the third TBUT procedure, the eyes were rinsed with PBS, and images were captured using a cobalt blue filter under the slit-lamp microscope to assess corneal epithelial damage indicated by areas of fluorescein staining. The corneas were graded based on fluorescein staining severity in four quadrants of the cornea, as outlined above.

### Tear Drying Time

To obtain a tear sample from the ocular surface, 1 µL PBS solution was instilled onto the ocular surface, and thereafter, 2 µL was recovered from the medial canthus of each mouse eye. Care was taken to avoid damage to the ocular surface. The collected volume was carefully placed on a glass slide in a controlled environment. An equal volume of PBS was placed next to the tear sample and used as an internal control. The time taken for the tear to spontaneously dry until complete dehydration was recorded as tear drying time in seconds. Values were normalized to the drying time of the PBS control.

### Phenol Red Thread Test

To carry out the phenol red thread test (Zone-Quick; Showa Yakuhin Kako Co., Ltd., Tokyo, Japan), mice were manually restrained without sedation, and 1 mm of the phenol red thread length was inserted into the temporal conjunctival fornix using fine curved-tip forceps for 15 seconds. The thread was then removed and the length measured with a ruler by a second investigator in a masked manner in an environment with controlled temperature and humidity. Three measurements were taken per mouse at least 5 minutes apart and the mean length plotted on the graph.

### Statistical Analysis

Statistical analysis was carried out using GraphPad Prism version 5 software package (GraphPad Software, San Diego, CA, USA) and Microsoft Excel Version 16.75 (Microsoft, Redmond, WA, USA). The difference between the two groups was compared by Student's *t*-test and between more than two groups by one-way ANOVA followed by Tukey's post hoc tests. *P* values of ≤ 0.05 were considered statistically significant. All values are presented as a standard deviation of the mean.

## Results

### *Has1^−/−^; Has3^−/−^* Mice Are Better Protected From Age-Associated Corneal Opacity

**Figure 2.**
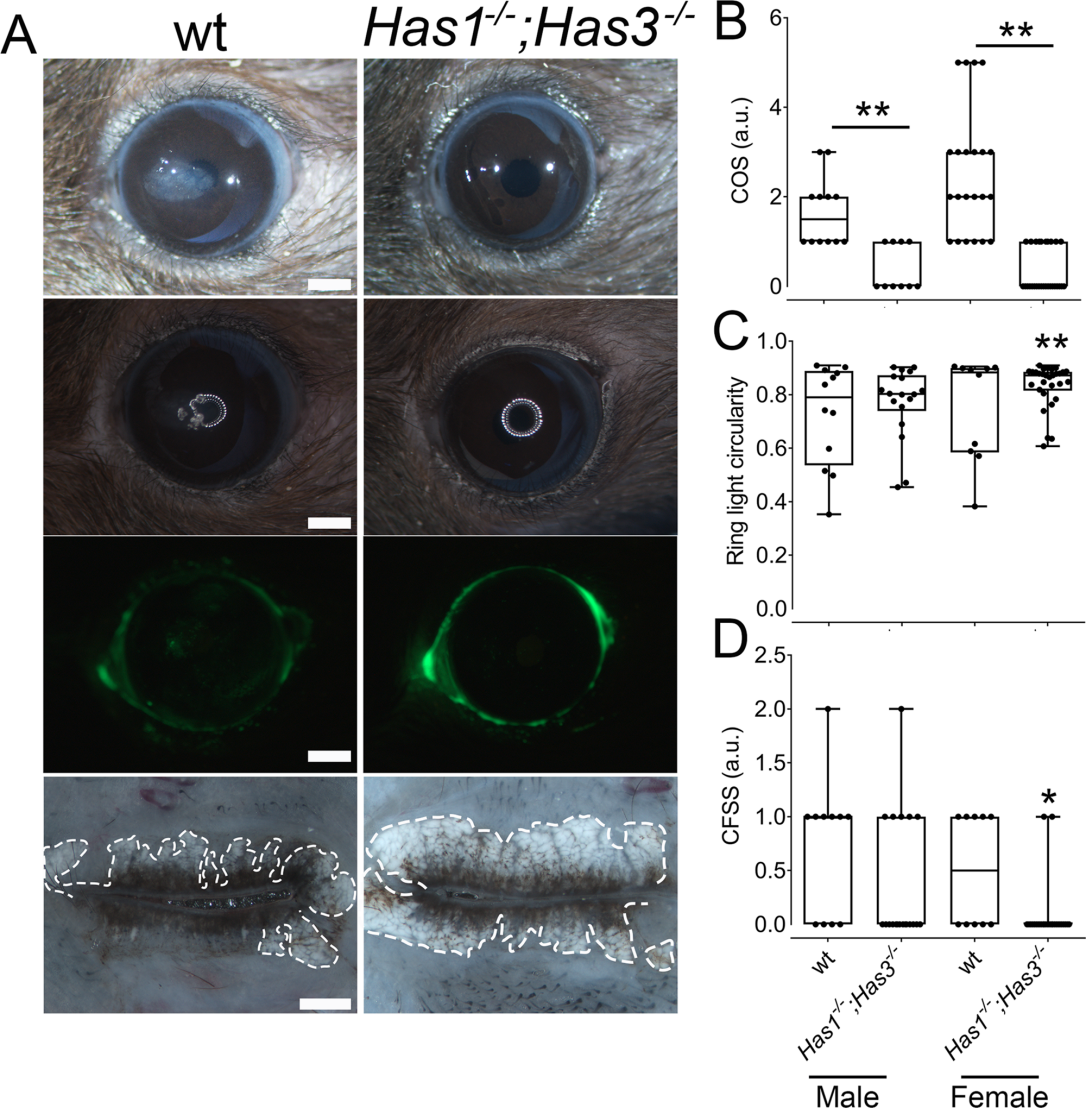
Aged *Has1^−/−^;* *Has3^−/−^* mice present less corneal opacity, increased corneal smoothness, reduced epithelial damage, and no MG atrophy. (**A**) Representative image of the ocular surface of aged wt and *Has1^−/−^;* *Has3^−/−^* mice captured under a stereomicroscope with white light (*top*), ring light (*second*), and after fluorescein administration using a GFP filter (*third panel*). Flat-mounted eyelid whole mounts were imaged under a stereomicroscope to evidence the MGs (*lowermost*). *Scale bar*: 1000 µm. (**B**) A COS was calculated from the white light–captured images of the eyes of male and female aged wt and *Has1^−/−^;* *Has3^−/−^* mice and plotted as a box plot. Each individual animal is represented as an individual point on the graph. (**C**) Ring light circularity index was calculated from the images of the reflected ring light onto the ocular surface of male and female aged wt and *Has1^−/−^;* *Has3^−/−^* mice using an in-house script and plotted as a box plot. Each individual animal is represented as an individual point on the graph. (**D**) A CFSS was calculated from the fluorescein-stained eyes of male and female aged wt and *Has1^−/−^;* *Has3^−/−^* mice using the GFP filter and plotted as a box plot. Each individual animal is represented as an individual point on the graph. *n* = 10. *P ≤ 0.05, **P ≤ 0.01.

The ocular surface of 2-year-old wt and *Has1^−/−^;* *Has3^−/−^* mice was assessed to study the effects of aging on corneal integrity, corneal opacity, and corneal surface irregularities. Images were captured with white light and ring light, followed by the administration of 0.1% fluorescein and imaged with a GFP filter under a stereomicroscope (Discovery.V12 Modular Stereo Microscope ; Carl Zeiss Microscopy LLC) (Figs. 2B, 2C). All aged wt mice presented some level of corneal opacity (Figs. 2A, 2B). The opacity observed in the wt mice ranged from a slight corneal opacity diffused throughout the cornea to severe opacity limited to one or two quadrants of the cornea, with a representative image provided (Fig. 2A). In contrast, *Has1^−/−^; Has3^−/−^* mice either did not present any corneal opacity or presented only slight corneal opacity diffused throughout the cornea, with a representative image provided (Fig. 2A). The COS was calculated, and for wt mice, the COS ranged from 1 to 2 for male wt mice and 1 to 3 for female wt mice (Fig. 2B). Although some female mice presented corneal opacity with increased severity, the difference in COS between the male and female groups did not reach statistical significance (Fig. 2B). Both male and female *Has1^−/−^; Has3^−/−^* mice had significantly less corneal opacity when compared to wt mice (Figs. 1A, 1B).

**Figure 3.**
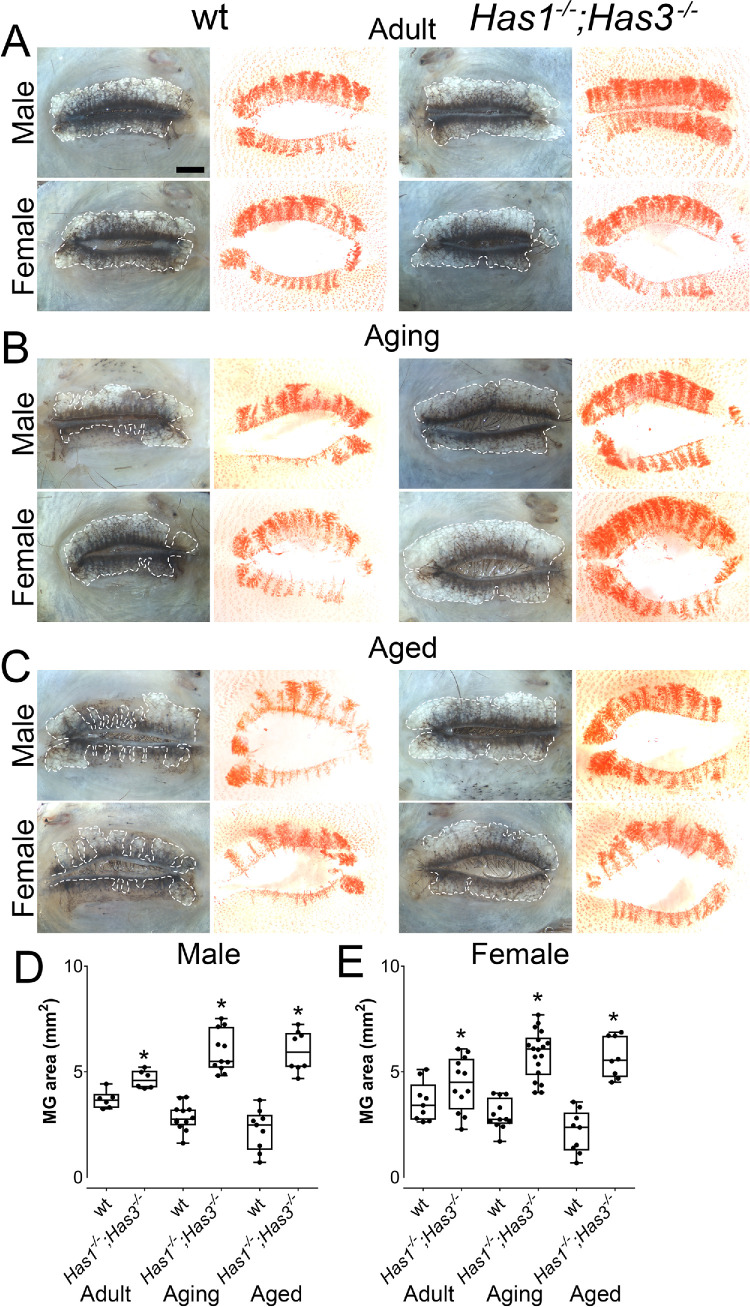
Qualitative lipid analysis using Sudan IV staining reveals larger amounts of lipoproteins, triglycerides, and other neutral lipids in aging and aged *Has1^−/−^;* *Has3^−/−^* mice MGs compared to wt mice. Representative images of eyelids showing whole-mount images of MGs of adult (**A**), aging (**B**), and aged (**C**) wt and *Has1^−/−^;* *Has3^−/−^* male and female mice, captured under white light before (*left*) and after (*right*) Sudan IV staining. A *white dashed line* was used to mark the outer perimeter of the MGs. All images were captured with the same magnification. The total MG area was quantified using ilastik version 1.4.0 for adult, aging and aged wt and *Has1^−/−^;* *Has3^−/−^* male (**D**) and female (**E**) mice. Each point of the graph indicates an individual mouse. *Scale bar*: 1000 µm.

The circularity of a ring light that is reflected from the ocular surface was previously proposed by the de Paiva group as a means to measure corneal smoothness in animal models of DED.^36^ Variations or defects in the smooth ocular surface cause distortions to the reflected ring light that can be quantified by measuring the circularity of the reflected light. Further, the clarity and stability of the reflected ring light can serve as an indicator of tear film stability, with a smooth, clear ring indicating a stable tear film and a distorted or blurry ring suggesting tear film instability. The ring circularity was calculated using an in-house script, with 1 presenting a perfect circle. Representative images of the reflected ring light are presented (Fig. 2A). *Has1^−/−^; Has3^−/−^* mice had a tendency to present a higher ring light circularity score when compared to wt mice, but reaching statistical significance only for the female mice (Fig. 2D). The eyelids of the mice were obtained and mounted for whole-mount imaging and the outer perimeter of the MGs traced with a white dashed line. Aged wt mice presented clear signs of MG atrophy and dropout, as previously shown^23^ (Fig. 2). Instead, *Has1^−/−^; Has3^−/−^* mice had limited to no signs of MG atrophy and dropout, presenting healthy MGs with no signs of MGD (Fig. 2).

To further assess the MGs of *Has1^−/−^; Has3^−/−^* and wt mice at different ages, eyelids were obtained, dissected, and processed for whole-mount imaging (Figs. 3A–C). The total area of the MGs was calculated for male and female mice at 8 weeks, 1 year, and 2 years (Figs. 3D, 3E). *Has1^−/−^; Has3^−/−^* mice presented a significantly larger MG area than wt mice at all time points analyzed (Figs. 3D, 3E). As mice age, they continue to undergo somatic growth, and as such, the overall size of the mouse progressively increases, which is accompanied by the proportional growth of tissues and organs. Thus, it can be noted that the length and width of the MGs of *Has1^−/−^; Has3^−/−^* and wt mice increase as they age from 8 weeks to 1 year. However, in the case of the wt mice, glands undergo MG atrophy and dropout; therefore, there is a slight drop in the overall MG area as the mice age from 8 weeks to 1 year. However, since the *Has1^−/−^; Has3^−/−^* mice do not present MG atrophy or dropout, their MGs are significantly larger at 1 year when compared to 8 weeks.

### Sudan IV Staining Reveals Larger Quantity of Lipoproteins, Triglycerides, and Other Neutral Lipids in *Has1^−/−^; Has3^−/−^* Mice Compared to wt Mice

To visualize the lipid content in the MGs, Sudan IV staining was used for eyelid whole-mount staining, as described in the Methods section. Sudan IV dye primarily stains triglycerides, along with other neutral lipids, including lipoproteins. Our Sudan IV staining-based qualitative analysis reveals that in wt mice, area and intensity of Sudan IV staining decreased with age, indicating atrophy and dropout-associated loss of glandular tissue and meibum production (Fig. 3), as reported previously.^23^ Interestingly, the staining area and intensity in the MGs in *Has1^−/−^;* *Has3^−/−^* mice were higher than wt at all ages. *Has1^−/−^; Has3^−/−^* mice presented an increase in meibum production at all ages when compared to the wt mice, and importantly, no significant loss in meibum production was observed in the *Has1^−/−^;* *Has3^−/−^* mice with aging (Fig. 3). These data further confirm our previous findings that *Has1^−/−^; Has3^−/−^* mice are resistant to developing ARMGD.^23^

### *Has1^−/−^; Has3^−/−^* Mice Produce Higher Amounts of Lipids Compared to wt Mice

**Figure 4.**
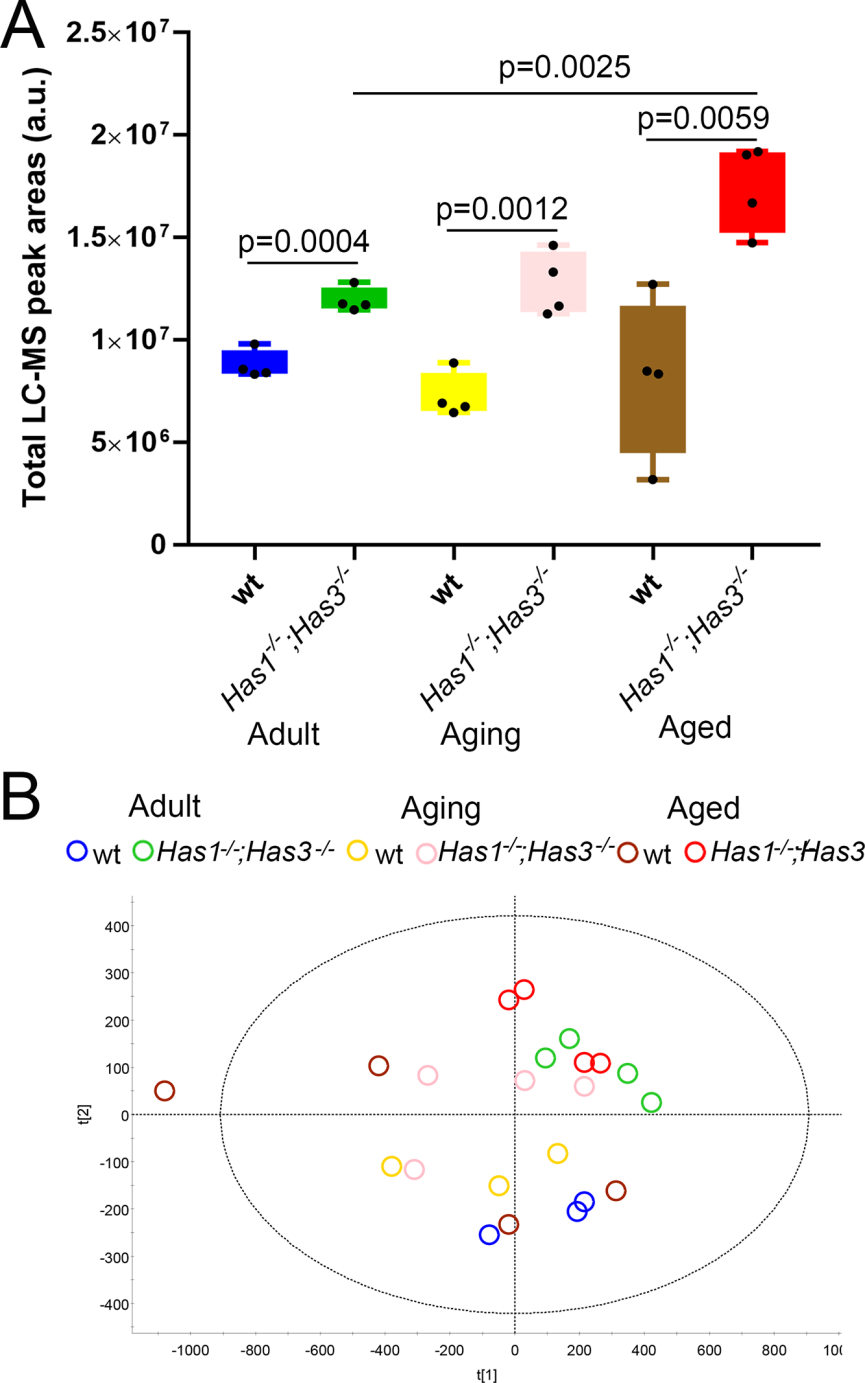
LC-MS analysis reveals a higher abundance of total lipids in the MGs of *Has1^−/−^; Has3^−/−^* mice at all ages, compared to wt mice. Untargeted PLS-DA analyses of the data demonstrated clear effects of altered HA expression on the MG lipid production from *Has1^−/−^;* *Has3^−/−^* mice and modest effects of aging on both age groups.

To provide quantitative data on lipid production in wt and *Has1^−/−^; Has3^−/−^* mice as they age, we carried out quantitative lipidomic analysis of meibum using LC-MS. Meibum was extracted from the MGs of *Has1^−/−^;* *Has3^−/−^* and wt adult, aging, and aged mice and subjected to quantitative lipid analysis using LC-MS, as described in the Methods section. Overall, the combined total peak area showed that *Has1^−/−^; Has3^−/−^* mice express increased levels of lipids when compared to wt mice at all time points, with a similar distribution to the total MG area (Fig. 4A). Thus, these data showed that *Has1^−/−^; Has3^−/−^* mice express more meibum than wt mice at all ages analyzed.

### Characterization of Lipid Composition in the Meibum of *Has1^−/−^; Has3^−/−^* and wt Mice

**Figure 5.**
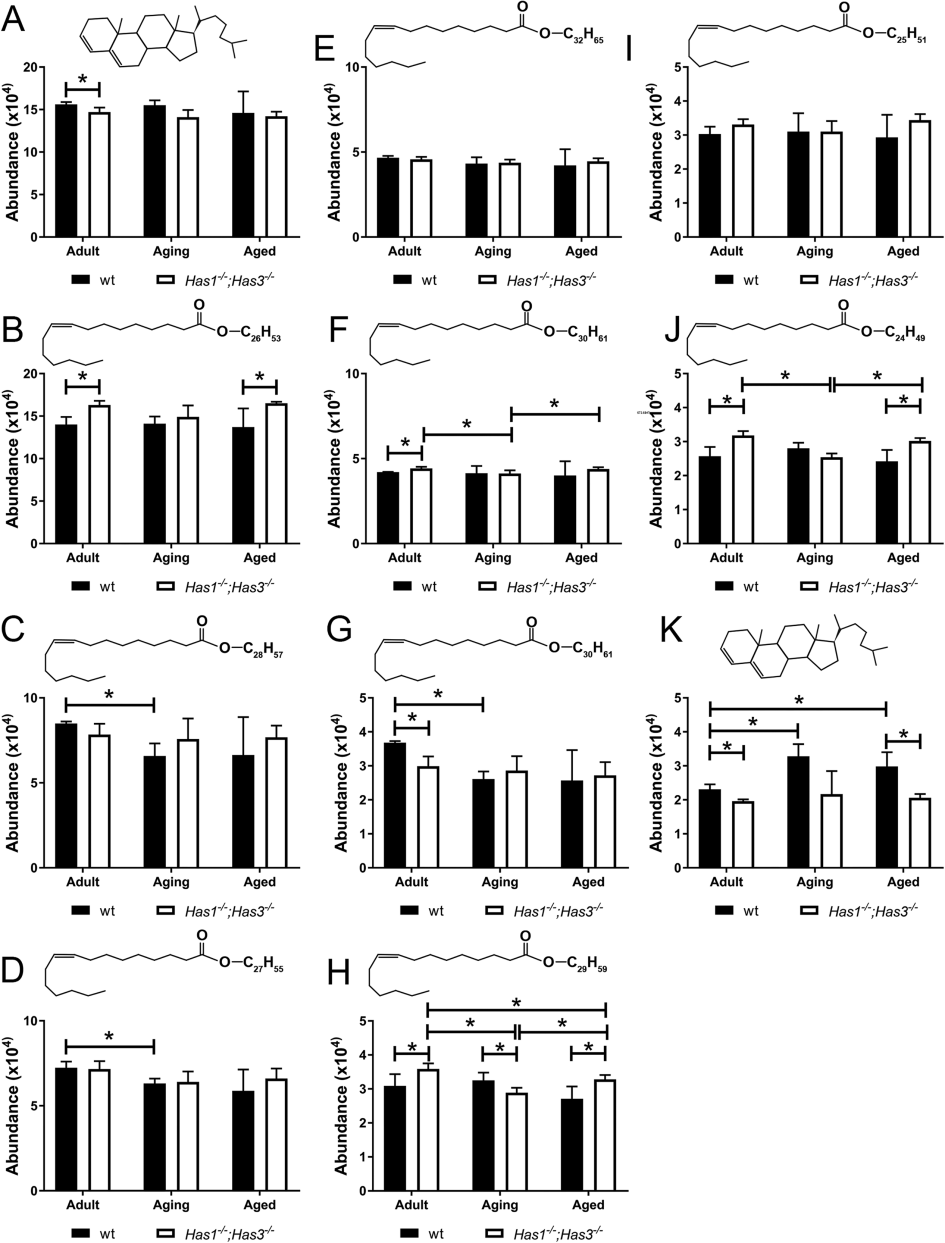
LC-MS analysis reveals differences in the abundance of certain lipid populations in the meibum of *Has1^−/−^; Has3^−/−^* mice compared to wt mice. Meibum isolated from the pooled samples of four eyelids of adult, aging, and aged wt and *Has1^−/−^; Has3^−/−^* mice was subjected to LC-MS analysis, and the most abundant lipid classes are shown (**A–K**) that included one CE (**A**), nine WEs (**B–J**), and one nonesterified cholesterol (**K**). The abundance of respective lipids across different ages is presented, with *black columns* representing abundance value from wt mice and *white columns* representing the ones from *Has1^−/−^;* *Has3^−/−^* mice. The *error bars* represent SEM. *P ≤ 0.05. The molecular structure on the top of each graph represents the lipid type identified for the specific *m/z* ratio, as summarized in Supplementary Table S1.

Untargeted lipid analyses of the six types of study samples were conducted using the PLS-DA approach (Fig. 4B). The analyses provided evidence of the effects of aging on both *wt* and *Has1^−/−^;* *Has3^−/−^* mice. Targeted LC-MS analysis of lipid populations in the meibum of wt and *Has1^−/−^;* *Has3^−/−^* mice revealed CEs, WEs, and nonesterified cholesterol as the most abundant lipid types. The amount of CEs, detected using their common analytical ion *m/z* 369.3522 (Fig. 5A), did not significantly change in either wt or *Has1^−/−^;* *Has3^−/−^* mice as they aged, but in the adult stage, the expression level of total CEs was significantly lower in *Has1^−/−^;* *Has3^−/−^* mice than in wt mice. Out of the nine WEs identified, C_48_H_92_O_2_ (diunsaturated C48:2) and C_44_H_84_O_2_ (diunsaturated; C44:2) with an *m/z* of 701.7691 and 645.6537, respectively, did not show any significant changes between the genotypes and ages (Figs. 5E, 5I). From the remaining WEs, C_42_H_82_O_2_ (monounsaturated; C42:1) with an *m/z* ratio of 619.6384 (Fig. 5B) and C_40_H_78_O_2_ (monounsaturated; C40:1) with an *m/z* ratio of 645.6537 were significantly higher in the *Has1^−/−^;* *Has3^−/−^* mice in the adult and aged stages when compared to wt mice. The C_46_H_88_O_2_ (diunsaturated; C46:2) with an *m/z* ratio of 673.6847 (Fig. 5F) had significantly higher expression in the *Has1^−/−^;* *Has3^−/−^* mice when compared to wt in the adult stage, with slight variations in the expression levels in the *Has1^−/−^;* *Has3^−/−^* mice as they aged. For C_45_H_88_O_2_ (monounsaturated; C45:1) with an *m/z* ratio of 661.6834 (Fig. 5G), the expression was significantly lower in *Has1^−/−^;* *Has3^−/−^* mice when compared to wt mice in the adult stage, but wt mice showed a significant decrease in expression of this WE as they aged, while there was no significant difference between the expression levels between the *Has1^−/−^;* *Has3^−/−^* and wt mice in the aging and aged stages. On the other hand, the WE C_41_H_80_O_2_ (monounsaturated; C41:1) with an *m/z* ratio of 605.6224 (Fig. 5H) was significantly higher in *Has1^−/−^;* *Has3^−/−^* mice in adult and aged stages compared to wt mice. The WEs C_44_H_86_O_2_ (monounsaturated; C44:1) (Fig. 5C), C_43_H_84_O_2_ (monounsaturated; C43:1) (Fig. 5D), and C_45_H_88_O_2_ (monounsaturated; C45:1) (Fig. 5G) with *m/z* ratios of 647.6690, 633.6533, and 661.6834, respectively, were significantly lower in aging wt mice compared to adult wt mice. However, there was no significant change in these lipid populations in the *Has1^−/−^;* *Has3^−/−^* mice as they aged. The WE C_40_H_78_O_2_ (monounsaturated; C40:1) with an *m/z* ratio of 591.6073 was expressed at significantly higher levels in the *Has1^−/−^;* *Has3^−/−^* mice when compared to the wt in the adult and aged stages (Fig. 5J). Finally, the nonesterified cholesterol with an *m/z* of 369.3524 (Fig. 5K) had significantly lower expression levels in the *Has1^−/−^;* *Has3^−/−^* mice compared to wt at all stages, namely, adult, aging, and aged. Curiously, the expression of this nonesterified cholesterol gradually increased in the wt mice as they aged, but the expression levels did not change in the *Has1^−/−^;* *Has3^−/−^* mice as they aged.

### Analysis of the Expression of Biosynthetic Enzymes Involved in Lipid Synthesis in *Has1^−/−^; Has3^−/−^* and wt

**Figure 6.**
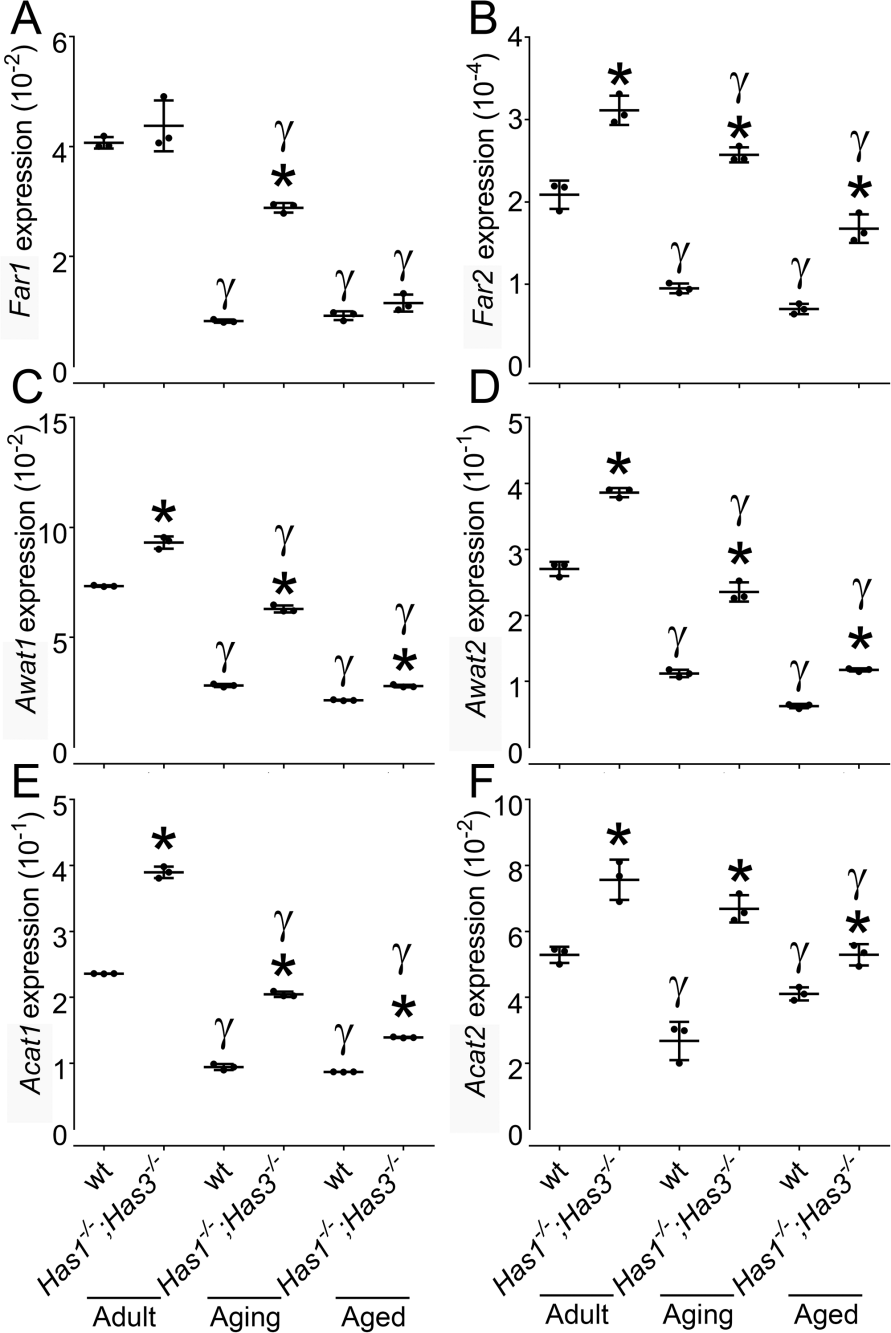
*Has1^−/−^; Has3^−/−^* mice present significantly higher expression of biosynthetic enzymes involved in lipid synthesis in MGs compared to wt mice. Eyelids were collected from wt and *Has1*^−/−^*; Has3*^−/−^ mice at 8 weeks, 1 year, and 2 years, and MGs were isolated after trimming away the tarsal plate. MGs pooled from eight mice were processed for quantitative RT-PCR to evaluate the expression of genes for meibum biosynthetic enzymes, namely, fatty acyl-coenzyme A reductases 1 (**A**) and 2 (**B**) (*Far1* and *Far2*), acyl-CoA wax alcohol acyltransferases 1 (**C**) and 2 (**D**) (*Awat1* and *Awat2*), and acyl-coenzyme A:cholesterol acyltransferases 1 (**E**) and 2 (**F**) (*Acat1* and *Acat2*). *P ≤ 0.05 when comparing the expression levels between wt and *Has1^−/−^;* *Has3^−/−^* mice at different ages, while γ represents *P* ≤ 0.05 when comparing the aging and aged mice of the same genotype to adult mice.

To further investigate how the lipid composition of meibum changes as wt and *Has1^−/−^;* *Has3^−/−^* mice age, we investigated changes in the expression of the genes involved in the biosynthesis of major lipid components of meibum, particularly WEs and CEs, using quantitative RT-PCR (Fig. 6). The selected genes included fatty acyl-coenzyme A reductases 1 and 2 (*Far1* and *Far2*), acyl-CoA wax alcohol acyltransferases 1 and 2 (*Awat1* and *Awat2*), and acyl-coenzyme A:cholesterol acyltransferases 1 and 2 (*Acat1* and *Acat 2*). *Has1^−/−^;* *Has3^−/−^* mice presented significantly higher expression of all these genes compared to wt mice at all ages, with the exception of *Far1*, which was only significantly increased in the aging *Has1^−/−^;* *Has3^−/−^* mice (Fig. 6). Overall, both wt and *Has1^−/−^;* *Has3^−/−^* mice presented a gradual and significant decrease in the expression of all the biosynthetic enzymes as they aged.

### *Has1^−/−^; Has3^−/−^* Mice Are Better Protected From BAC-Induced Dry Eye Symptoms, Compared to wt Mice

**Figure 7.**
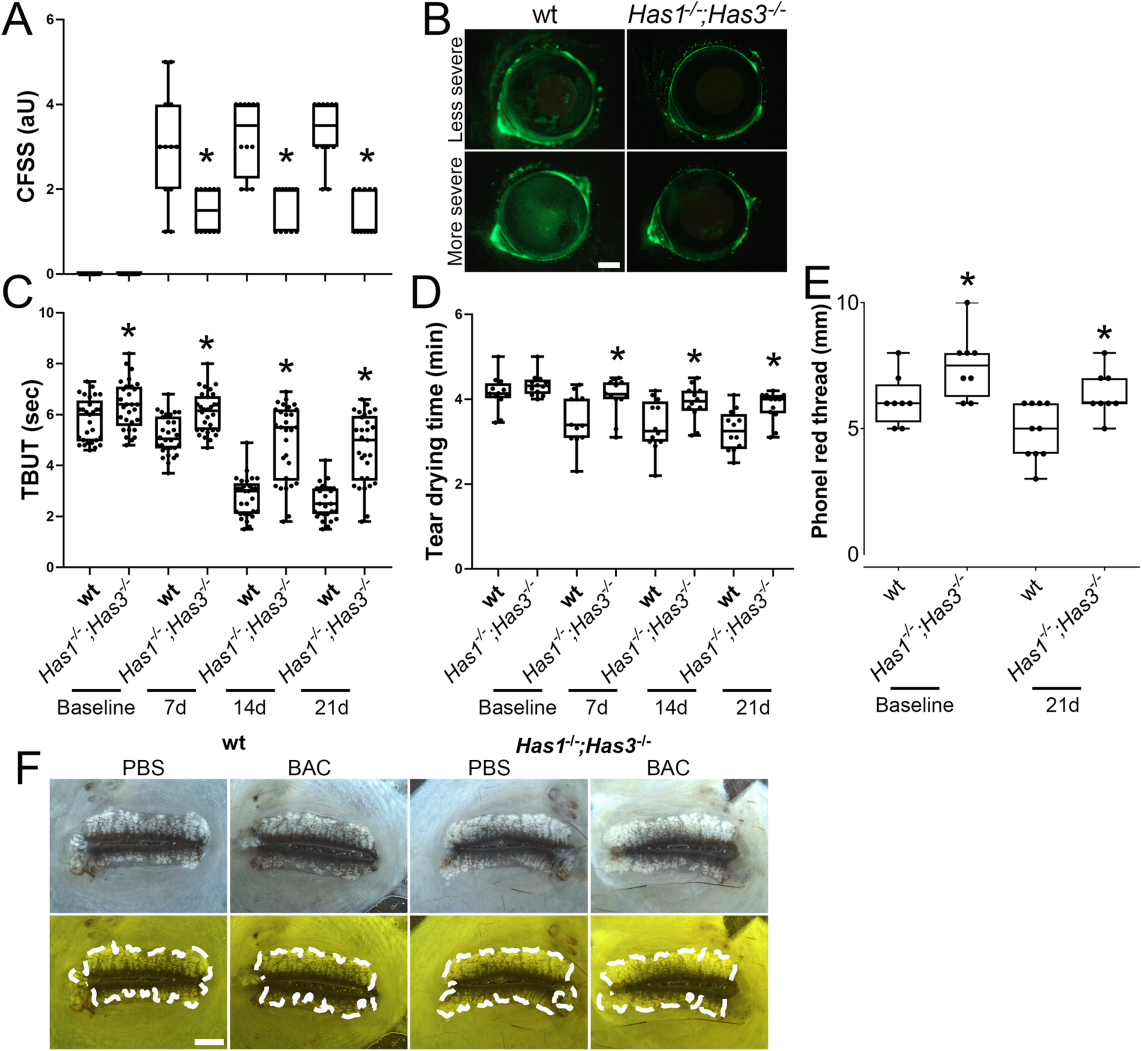
Adult *Has1^−/−^;* *Has3^−/−^* mice are protected from ocular surface damage following the induction of experimental BAC-induced dry eye when compared to wt. *Has1^−/−^;* *Has3^−/−^* and wt mice were subjected to experimental BAC-induced dry eye via the topical administration of 0.1% BAC twice daily for 3 weeks. (**A**) CFSS was assessed in images acquired from the ocular surface after fluorescein administration in a blinded manner and a corneal fluorescein staining score attribute. Data are presented as a box plot, and each individual point represents an individual animal. (**B**) Representative images of a more severe and less severe case of corneal fluorescein staining for *Has1^−/−^;* *Has3^−/−^* and wt mice. (**C**) The TBUT was calculated in a blinded manner for *Has1^−/−^;* *Has3^−/−^* and wt mice at the end of each week. Data are presented as a box plot, and each individual point represents an individual animal. (**D**) The tear drying time was calculated in a masked manner for *Has1^−/−^;* *Has3^−/−^* and wt mice at the end of each week. (**E**) The tear volume was measured using the phenol red thread test by inserting 1 mm of the thread into the temporal conjunctival fornix of a manually restrained mouse for 15 seconds. Data are presented as a box plot, and each individual point represents an individual animal. (**F**) At the end of the third week, the animals were euthanized, and the eyelids were obtained and processed for whole-mount imaging. Representative whole -mount images of MGs captured under white light and under the GFP filter from the control and BAC-treated wt and *Has1^−/−^;* *Has3^−/−^* mice are shown. The outer perimeter of the MG is traced with a *white dashed line*. *Scale bar*: 1000 µm. *P ≤ 0.05.

Our combined data demonstrate that adult *Has1^−/−^;* *Has3^−/−^* mice present larger MGs that produce an increased amount of meibum with a slightly different composition when compared to wt mice. To verify whether these enlarged glands that produce increased amounts of meibum with a slightly different composition compared to wt mice can protect *Has1^−/−^;* *Has3^−/−^* mice from DED, we subjected adult *Has1^−/−^;* *Has3^−/−^* and wt mice to the BAC DED model. BAC is a cationic surfactant that functions as a detergent that is often found in ophthalmic solutions as a preservative with concentrations typically ranging from 0.004% to 0.02%. BAC disrupts the tear film by breaking down the lipid layer, increasing tear evaporation and leading to tear film instability. Excessive use of BAC can lead to epithelial damage to the cornea and conjunctiva caused by the disruption of the lipid bilayer of epithelial cell membranes. In our model, we selected a 0.1% BAC dose twice daily for 3 weeks to attempt to limit the damage to that caused by disruption of the tear film. *Has1^−/−^;* *Has3^−/−^* and wt mice were analyzed before starting the BAC model as a baseline and then at the end of each week for 3 weeks. *Has1^−/−^;* *Has3^−/−^* mice presented significantly less corneal damage, as assessed via fluorescein staining, when compared to wt mice at all time points analyzed (Figs. 7A, 7B). *Has1^−/−^; Has3^−/−^* mice presented a CFSS ranging between 1 and 2, whereas wt mice presented a CFSS ranging from 1 to 5, with most mice between 3 and 4 (Fig. 7A). *Has1^−/−^; Has3^−/−^* mice also presented significantly higher TBUT and tear drying time when compared to wt mice at all time points analyzed (Figs. 7C, 7D). *Has1^−/−^; Has3^−/−^* mice also presented increased tear volume when compared to wt mice, as measured by the phenol red thread test (Fig. 7E). Thus, *Has1^−/−^; Has3^−/−^* mice were protected from ocular surface damage when subjected to the BAC dry eye model when compared to wt mice. The eyelids were obtained, flat-mounted, and imaged to ascertain that all mice had healthy MGs and that the short-term topical use of BAC had not caused any damage to the MGs. All mice included in the BAC dry model presented healthy MGs, with representative images presented in Figure 7E. Thus, short-term administration of BAC did not cause any direct damage to the MGs.

## Discussion

Our previous work demonstrated that an HA-rich matrix is present surrounding the MG and within the tarsal plate in mice.^22^^,^^23^ With aging, there is a significant loss of HA surrounding the MG and within the tarsal plate that is associated with MG atrophy.^23^ Furthermore, the induction of a loss of HA surrounding the MG and within the tarsal plate using a chemical inhibitor that inhibits HA synthesis triggers MG atrophy.^23^ Interestingly, *Has1^−/−^; Has3^−/−^* mice upregulate *Has2* through a compensatory mechanism and, in turn, present precocious MG development, and adult mice have enlarged glands when compared to wt mice.^22^ Curiously, *Has1^−/−^; Has3^−/−^* mice do not present a loss of HA with aging and thus do not present MG atrophy or ARMGD. Instead, their MGs continue to increase in size as they age, and by 2 years they present a 4-fold increase in MG area when compared to wt mice.^23^ The present study aimed to characterize the composition of the meibum produced by *Has1^−/−^; Has3^−/−^* mice as they age compared to wt mice. Furthermore, we functionally characterized the meibum by verifying whether the meibum can stabilize the tear film as the mice age and whether they are protected from developing DED.

Aging causes significant changes in the structure and function of the ocular surface that can significantly affect vision, such as a functional loss of lacrimal glands (LGs)^37^^,^^38^ and MG atrophy and dropout.^16^ Functional changes to LGs and MGs can lead to changes in the amount and composition of tears, which directly affect ocular health.^39^ In fact, several studies have clearly described the changes in meibum composition that occur with aging, which can lead to changes in the appearance and consistency of meibum.^40^^,^^41^ Even slight variations in the composition of meibum can lead to significant physical and physiological changes and failure to stabilize the tear film, leading to dry eye symptoms.^42^ Meibum is primarily composed of lipids^43^ and some proteins.^44^ In our study, the top 10 lipid populations identified were cholesterol, CEs, monounsaturated WEs with carbon chains ranging from 40 to 46 carbons in length, diunsaturated WEs with carbon chains ranging from 46 to 48 carbons in length, and nonesterified cholesterol. A previous study found no significant changes in lipid biosynthetic machinery via microarray-based mRNA expression analysis between 2- and 32-month-old mice.^35^ We previously found that some atrophic MGs are already evident by 32 weeks, although mice have significantly more MG atrophy after 1 year. Our LC-MS–based lipidomic analysis of meibum revealed that the meibum of wt mice has some changes in lipid composition by 1 and 2 years. Specifically, there was a decrease in monounsaturated WEs, including C_44_H_86_O_2_ (*m/z* of 647), C_45_H_88_O_2_ (*m/z* of 661), and C_43_H_84_O_2_ (*m/z* of 633) with aging, and an increase in nonesterified cholesterol (*m/z* of 369). Overall, *Has1^−/−^; Has3^−/−^* mice presented a similar lipid profile to wt mice, with few notable differences. *Has1^−/−^; Has3^−/−^* mice presented significantly increased levels of monounsaturated WE (C_42_H_82_O_2_, *m/z* of 619), monounsaturated WE (C_40_H_78_O_2_, *m/z* of 591), and monounsaturated WE (C_41_H_80_O_2_, *m/z* of 605), as well as decreased monounsaturated WE (C_45_H_88_O_2_, *m/z* of 661) and nonesterified cholesterol expression compared to wt mice. Interestingly, *Has1^−/−^; Has3^−/−^* mice presented fewer age-related changes in the lipid composition of their meibum when compared to wt mice, indicating that the composition of meibum in *Has1^−/−^; Has3^−/−^* mice does not have significant age-related changes. Overall, there was a significant increase in the total amount of lipids produced in *Has1^−/−^; Has3^−/−^* mice when compared to wt mice, confirming that *Has1^−/−^; Has3^−/−^* mice produce significantly more meibum than wt mice. Previous studies have identified that murine meibum is mainly composed of WE, CE, (O-acyl)-ω-hydroxy fatty acids (OAHFAs) and their esters, free fatty acids (FFAs), acylglycerols, cholesterol (Chl), diacylated diols (diacylated α,ω-diol), and a smaller amount of other polar and nonpolar lipids.^43^ In humans, studies have shown that with aging, the hydrocarbon chain order in meibum decreases from ∼48% of *trans* rotamers at birth to ∼30% *trans* rotamers by 85 years of age.^45^ The phase transition temperature of meibum also decreases from ∼31°C at birth to ∼27°C by 90 years of age.^45^ Age-associated changes in human meibum physical properties have been attributed to compositional changes in meibum lipids.^45^ Furthermore, neutral and polar lipid profiles of human meibum have been shown to change with age.^46^ Suzuki et al.^41^ reported a significant increase in polar lipids Chl, OAHFA, and FFA; a significant decrease in nonpolar lipids (e.g., CEs); and no change in WE with age. Additionally, with age, the relative amount of CH_3_, C=C bonds, and degree of oxidation increases.^47^^,^^48^ An age-associated increase in the aldehyde-to-hydroperoxide ratio suggests a progressive increase in oxidation of the meibum.^47^ In contrast, Yeotikar et al.^49^ reported no significant age-associated changes in major lipid classes of human meibum using nano-electrospray ionization tandem mass spectrometry. Similarly, Butovich and Suzuki^40^ did not observe significant age-associated changes in lipid esterification, elongation, length of carbon chain, and degree of saturation in human meibum using LC-MS and MS-MS either. The only lipid class that showed statistically significant changes was diacylated α,ɷ-diols (DiAD).^40^ The reason for varied reports on the effect of aging on meibum lipid composition could be attributed, at least in part, to varying methods of meibum collection, storage and analysis, and incidental contamination.^50^^,^^51^ While our study provides valuable insights into age-associated changes in the composition, distribution, and function of meibum in mice with altered HA expression, there are some considerations to discuss while interpreting the results of our study. For example, in the analysis of total lipids from tarsal plates using LC-MS, there is a possibility of contamination of lipids from other cells/tissues. However, the discovered differences between the meibum samples that were expressed directly from the eyelids and those extracted from the tarsal plates with organic solvents were minimal (Fig. 1) and, thus, inconsequential for the purpose of this study. Moreover, the differences in the relative balance of different lipid populations between the experimental groups were far less pronounced than the total amount of lipids produced by MGs, which is an important factor to consider while interpreting the physiological impact of the findings. Also, for the purpose of comparative analysis, the same number/quantity of excised tarsal plates per experimental group was used as a standard approach to minimize the difference(s) in the amount of sample used. This approach is far more reproducible than the physical expression of meibum, which cannot guarantee full expression of the secretion from the glands. Moreover, for individual lipid classes (Fig. 4), it must be noted that the lipids from MGs (i.e., extremely long chain [ELC] WEs and CEs) are primarily synthesized by meibocytes. Only minimal amounts are produced by sebocytes, and those are not of the ELC variety since they are much shorter in length.

WEs and CEs have been reported as major lipid classes of the meibum, in both humans and mice.^43^^,^^52^ Since, WEs and CEs were also identified in our study as the most abundant lipid classes in meibum, the expression of enzymes involved in the biosynthesis of these lipids was analyzed by quantitative PCR. FAR1 and FAR2 catalyze the reduction reaction for synthesizing fatty alcohol from fatty acyl-CoA.^53^ AWAT1 and AWAT2 catalyze the formation of ester bonds between fatty alcohol and fatty acyl-CoA to form WEs.^54^ ACAT1 and ACAT2 catalyze the synthesis of CEs using long-chain fatty acyl-CoA and cholesterol as substrates.^55^ We observed a significant age-related decline in the expression of all biosynthetic enzymes analyzed (i.e., *Far 1* and *2*, *Awat 1* and *2*, and *Acat 1* and *2* ) in both wt and *Has1*^−/−^*; Has3*^−/−^ mice, indicating that, with age, there is a significant decrease in the expression of enzymes involved in the biosynthesis of WEs and CEs within MGs. However, there was significantly higher expression of the biosynthetic enzymes in *Has1*^−/−^*; Has3*^−/−^ mice compared to wt mice at all time points analyzed. The fact that there was an observed decrease in the expression of biosynthetic enzymes in *Has1*^−/−^*; Has3*^−/−^ mice with aging was surprising, since we did not observe a decrease in meibum staining over time, there was an increase in total LC-MS peak area indicating an increase in total meibum, and there was a significant increase in MG area with aging in the *Has1*^−/−^*; Has3*^−/−^ mice.^23^ Since the expression levels of the biosynthetic enzymes are normalized based on the expression of housekeeping genes for which expression levels are relative to the total number of cells, we could speculate that the expression levels of the biosynthetic enzymes decrease over time per cell. However, since MGs are significantly larger in size in *Has1*^−/−^*; Has3*^−/−^ mice, overall, they produce higher levels of meibum than young adult *Has1*^−/−^*; Has3*^−/−^ and wt mice.

The histochemical staining of MGs using Sudan IV further revealed that *Has1*^−/−^*; Has3*^−/−^ mice produce higher amounts meibum compared to wt at all ages studied. Curiously, three forms of MGD exist: obstructive MGD, hyposecretory MGD, and hypersecretory MGD.^16^^,^^56^ Thus, studies have shown that hyperproduction and secretion of meibum can also disrupt tear film stability and cause DED.^56^ However, little is known about hypersecretory MGD, and studies are still needed to establish whether changes in meibum composition occur with hypersecretory MGD that could also contribute to the failure to stabilize the tear film. Given the significant increase in the size of MGs, the increase in the amount of meibum produced, and changes in the composition of the meibum in *Has1*^−/−^*; Has3*^−/−^ mice compared to wt mice, we further carried out functional studies to investigate whether the meibum produced in *Has1*^−/−^*; Has3*^−/−^ mice can successfully stabilize the tear film and promote ocular surface homoeostasis. Importantly, substantial prior work has established that changes in the composition of the meibum have a direct effect on the physical and physiological properties of the tear film, directly affecting ocular surface homeostasis. Interestingly, we found that a significant proportion of unchallenged aged wt mice developed corneal opacity and a loss of the smooth ocular surface. Thus, as part of the aging process, wt mice developed corneal opacity, and by 2 years, almost all mice had some level of corneal clouding. In stark contrast, *Has1*^−/−^*; Has3*^−/−^ mice presented limited to no corneal clouding and a smooth ocular surface at 2 years. Previous studies have reported that a high prevalence of corneal opacity is observed in aged humans^57^ and mice.^58^ Furthermore, the incidence of corneal irregularity and roughness significantly increases with age.^36^ Although the direct cause of the corneal opacity associated with aging remains unknown, studies have shown that age-associated changes in the extracellular matrix of corneal stroma occur.^59^ Corneal collagen fiber degeneration, an increase in glycation and advanced glycation end products, a decrease in corneal collagen interfibrillar spacing, and nonenzymatic cross-linking of the corneal stroma collagen fibrils are some of the age-related changes in corneal ECM that have been shown to affect corneal transparency and smoothness with aging.^59^ Our study indicates that in wt mice, DED that occurs as a consequence of ARMGD could contribute to the corneal clouding that occurs with aging. Thus, persistent inflammation associated with ARMGD in wt mice could lead to corneal scarring, evidenced by the loss of transparency and smoothness in aged mice. Interestingly, since *Has1*^−/−^*; Has3*^−/−^ mice do not present ARMGD, they would be protected from DED-associated inflammation and thus are protected from the age-associated corneal clouding that occurs in wt mice. It is important to note that our lab has previously shown that HA is highly expressed in the limbal stem cell niche and that HA is essential for maintaining limbal epithelial stem cells.^24^ Furthermore, HA is upregulated within the cornea following injury and is an integral component of the provisional matrix.^24^^,^^60^^–^^63^ Our prior work shows that although *Has1*^−/−^*; Has3*^−/−^ mice present healthy corneas if left unchallenged, they have delayed wound healing, increased inflammatory cell infiltration, increased corneal scarring, and the development of chronic corneal epithelial erosions following alkali burn when compared to wt mice.^24^ Thus, the fact that aged *Has1*^−/−^*; Has3*^−/−^ mice presented clear healthy corneas further supports the notion that they have a healthier tear film when compared to wt mice.

To further investigate whether the meibum produced by *Has1*^−/−^*; Has3*^−/−^ mice can successfully stabilize the tear film and protect the ocular surface against the deleterious effects of DED, we subjected adult wt and *Has1*^−/−^*; Has3*^−/−^ mice to an experimental model of BAC-induced dry eye. BAC, a quaternary ammonium compound having detergent-like properties, is a common preservative in ophthalmic preparations at a concentration range of 0.005% to 0.02%.^64^^,^^65^ As a detergent, it disrupts the lipid layer of the tear film, leading to DED symptoms. However, studies have shown that BAC can also damage the corneal epithelial cell membrane by dissolving intercellular junctions, resulting in epithelial cell loss, infiltration of cytokines, and conjunctival metaplasia.^66^^,^^67^ Zhang et al.^68^ previously showed that 0.2% BAC induced severe corneal epithelial defects, while 0.05% and 0.1% BAC induced significantly less corneal damage. Similarly, in our study, 0.2% BAC instilled twice daily onto the ocular surface led to severe epithelial damage in both wt and *Has1*^−/−^*; Has3*^−/−^ mice, albeit significantly less severe in the *Has1*^−/−^*; Has3*^−/−^ mice (data not shown). Our data indicate that this excessive damage was likely caused in part by direct damage of BAC to epithelial cells by damaging the cell membrane. In contrast, 0.05% BAC instilled twice daily did not induce significant DED symptoms in either wt or *Has1*^−/−^*; Has3*^−/−^ mice (data not shown). Therefore, 0.1% BAC instilled onto the ocular surface twice daily was selected for our experimental model of DED since our data indicated it destabilized the tear film and led to corneal epithelial damage, both hallmarks of DED, and the symptoms gradually increased in severity each week.^69^^,^^70^ Using this model, *Has1*^−/−^*; Has3*^−/−^ mice presented significantly decreased CFSS, increased TBUT, increased tear volume, and increased tear drying time compared to wt mice, indicating they were protected from experimentally induced DED. However, it is important to note that, as a limitation of this model, 0.1% BAC could have caused direct damage to corneal epithelial cells and/or led to a loss in goblet cell density in addition to damage caused by the experimentally induced DED. Taken together, our data indicate that *Has1*^−/−^*; Has3*^−/−^ mice have enlarged MGs that produce increased amounts of meibum with a slightly different composition to wt mice. The meibum produced by *Has1*^−/−^*; Has3*^−/−^ mice successfully promotes ocular surface homeostasis and protects against DED.

Previous work by our lab showed that a loss of HA in aging wt mice leads to MG atrophy and ARMGD. In contrast, *Has1^−/−^; Has3^−/−^* mice upregulate *Has2* expression and express increased levels of HA surrounding MGs and in the tarsal plate that do not decrease as they age.^21^^,^^22^ The present study aimed to characterize the lipid composition of the meibum produced by *Has1^−/−^; Has3^−/−^* mice compared to wt mice and establish whether it can successfully stabilize the tear film. Herein, we demonstrated that, indeed, *Has1^−/−^; Has3^−/−^* mice produce an increased amount of meibum when compared to wt mice, with a slightly distinct lipid composition. Importantly, the meibum produced by *Has1^−/−^; Has3^−/−^* mice successfully stabilizes the tear film and protects the mice from the age-associated corneal damage observed in wt mice. Thus, we can speculate that preventing the age-related loss of HA within the tarsal plate and surrounding MGs could serve as a potential therapeutic approach for preventing MG atrophy and supporting MG homeostasis. Further studies are needed to explore the potential therapeutic avenues for preventing the loss of HA surrounding MGs and within the tarsal plate, with the goal of preventing MG atrophy.

## Supplementary Material

Supplement 1
