# Supplementary material for: Comparative Analysis of Age-Associated Changes in Meibum Composition, Distribution, and Function in Mice With Altered Hyaluronan Expression
Source: Invest Ophthalmol Vis Sci. 2025 Jul 30;66(9):72. doi: 10.1167/iovs.66.9.72 (PMC12315927; doi:10.1167/iovs.66.9.72)
Supplement: Supplement 1 [file iovs-66-9-72_s001.pdf]

Supplementary Table 1: Most abundant lipids identified in the meibum of different age groups of wt and *Has1*$^{-/-}$;*Has3*$^{-/-}$ mice using LC-MS analysis

Figure no.	m/z	Type	Molecular formula	Molecular structure
4A	369.3522	CE	a common analytical ion of Chl (M – H_2O + H)+ and all cholesteryl esters (M – fatty acid + H)+	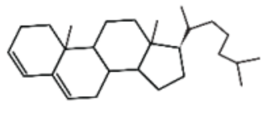
4B	619.6384	WE	a (M + H)+ ion of $C_{42}H_{82}O_2$ (monounsaturated; C42:1)	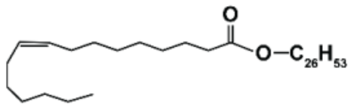
4C	647.669	WE	a (M + H)+ ion of $C_{44}H_{86}O_2$ (monounsaturated; C44:1)	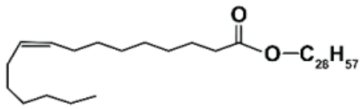
4D	633.6533	WE	a (M + H)+ ion of $C_{43}H_{84}O_2$ (monounsaturated; C43:1)	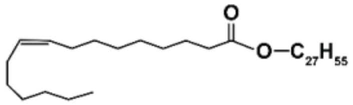
4E	701.716	WE	a (M + H)+ ion of $C_{48}H_{92}O_2$ (diunsaturated; C48:2)	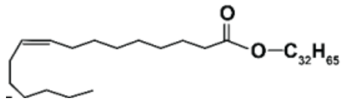
4F	673.6847	WE	a (M + H)+ ion of $C_{46}H_{88}O_2$ (diunsaturated; C46:2)	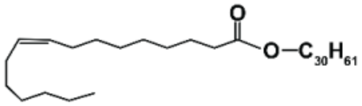
4G	661.6834	WE	a (M + H)+ ion of $C_{45}H_{88}O_2$ (monounsaturated; C45:1)	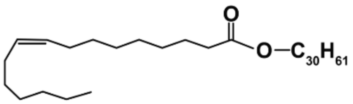
4H	605.6224	WE	a (M + H)+ ion of $C_{41}H_{80}O_2$ (monounsaturated; C41:1)	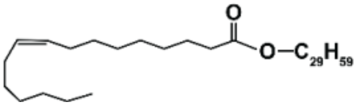
4I	645.6537	WE	a (M + H)+ ion of $C_{44}H_{84}O_2$ (diunsaturated; C44:2)	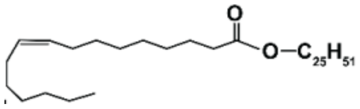

4J	591.607	WE	a (M + H)+ ion of $C_{40}H_{78}O_2$ (monounsaturated; C40:1)	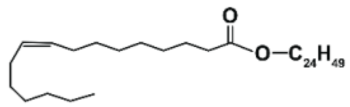
4K	369.3524	Non-esterified Chl	Signal of both cholesterol and cholesteryl esters	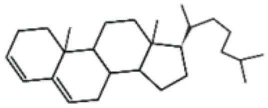
